# Quantum Imaging of Ferromagnetic van der Waals Magnetic Domain Structures at Ambient Conditions

**DOI:** 10.1021/acsami.5c16352

**Published:** 2025-11-06

**Authors:** Bindu, Amandeep Singh, Amir Hen, Lukas Drago Ćavar, Sebastian Maria Ulrich Schultheis, Shira Yochelis, Yossi Paltiel, Andrew F. May, Angela Wittmann, Mathias Kläui, Dmitry Budker, Hadar Steinberg, Nir Bar-Gill

**Affiliations:** † Institute of Applied Physics, 26742 The Hebrew University of Jerusalem, Jerusalem 9190401, Israel; ‡ Institute of Physics, 9182 Johannes Gutenberg University Mainz, 55128 Mainz, Germany; ¶ Materials Science and Technology Division, 6146 Oak Ridge National Laboratory, Oak Ridge, Tennessee 37831, United States; § Johannes Gutenberg-Universität Mainz, 55128 Mainz, Germany; ∥ The Racah Institute of Physics, The Hebrew University of Jerusalem, Jerusalem 9190401, Israel; ⊥ 530590 Helmholtz-Institut Mainz, GSI Helmholtzzentrum für Schwerionenforschung, 55128 Mainz, Germany; # Department of Physics, University of California, Berkeley, California 94720, United States; @ The Center of Nano-Science and Nanotechnology, The Hebrew University of Jerusalem, Jerusalem 9190401, Israel

**Keywords:** ferromagnetism, 2D van der Waals magnet, magnetic imaging, nitrogen vacancy center, autocorrelation, phase transition

## Abstract

Recently discovered 2D van der Waals magnetic materials, and specifically iron–germanium–telluride (Fe_5_GeTe_2_), have attracted significant attention both from a fundamental perspective and for potential applications. Key open questions concern their domain structure and magnetic phase transition temperature as a function of sample thickness and external field, as well as implications for integration into devices such as magnetic memories and logic. Here we address key questions using a nitrogen-vacancy center based quantum magnetic microscope, enabling direct imaging of the magnetization of Fe_5_GeTe_2_ at submicrometer spatial resolution as a function of temperature, magnetic field, and thickness. This quantum imaging technique provides noninvasive, high-sensitivity measurements with high spatial resolution under ambient conditions, making it particularly well suited for probing 2D magnets. We employ spatially resolved measures, including magnetization variance and cross-correlation, and find a significant spread in transition temperature yet with no clear dependence on thickness down to 15 nm. We also identify previously unknown stripe features in the optical as well as magnetic images, which we attribute to modulations of the constituting elements during crystal synthesis and subsequent oxidation. Our results suggest that the magnetic anisotropy in this material does not play a crucial role in their magnetic properties, leading to a magnetic phase transition of Fe_5_GeTe_2_ which is largely thickness-independent down to 15 nm. Our findings could be significant in designing future spintronic devices, magnetic memories, and logic with 2D van der Waals magnetic materials.

## Graphical abstract

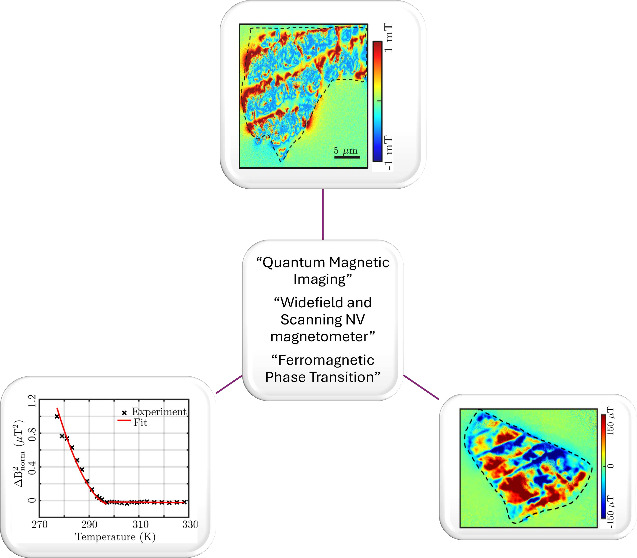

## 1 Introduction

In recent years, the advent of 2D van der Waals (vdW) materials that can be exfoliated down to one or few layers has opened new frontiers encompassing material science, fundamental physics, and novel applications.^1,2^ Magnetic vdW materials form an important subclass of this family,^3^ posing significant open questions, both fundamental and applied. For example, unknown aspects of these materials are associated with the structure of magnetization in thin flakes, thickness dependence of their magnetic properties, and implications of interfacial and structural properties on magnetization.

Studies of magnetic vdW materials have addressed some of these questions, spanning different materials and different experimental techniques. These include anomalous Hall effect measurements,^4^ reflective magnetic circular dichroism,^5^ magneto-optical Kerr effect,^6^ and integration of magnetic barriers into tunneling devices,^7^ addressing the behavior of magnetization, magnetic domains, and Curie temperature (T_C_) in recently discovered 2D vdW magnets. Moreover, magnetic imaging techniques have been employed to obtain spatially resolved information related to the magnetic properties of such materials.^8–10^ Specifically, nitrogen-vacancy (NV) center based magnetic imaging modalities, both wide field imaging^11^ and scanning NV center magnetometry (SNVM)^9^ have been employed in this context.

In this work, we focus on the recently identified vdW ferromagnet Fe_5_GeTe_2_,^12,13^ due to its near-room-temperature T_C_ ^14^ and its potential relevance for future research and applications. Bulk and thin layer studies of this material have characterized its magnetization structure and T_C_,^13^ current-induced domain-wall motion,^15^ and ferromagnetic resonance.^16^

Here we go beyond previous results and use NV center based Quantum Diamond Microscope­(QDM) to investigate the impact of interfacial and anisotropy effects on vdW magnets. We study the magnetization structure of FGT for various flake thicknesses, as a function of external magnetic field and temperature. Through spatial magnetic imaging of selected flakes, we identify a large spread in T_C_ regardless of flake thickness, down to 15 nm. It is worth noting that in few layer regime for FGT^4^ and cobalt doped FGT^8^ the decrease in T_C_ is relatively more pronounced. We further find previously unknown magnetic structures associated with crystallographic features in the material. These results provide important information on the magnetic properties of FGT and vdW magnets in general, highlighting the relevance of interfacial effects on magnetic behavior.

### 1.1 Quantum Magnetic Sensing

Quantum sensing in general is a highly developed field, based on the premise of using a quantum system to sense physical quantities such as magnetic fields, temperature and more.^17^ A broad range of quantum sensor implementations have been realized, including neutral atoms, trapped ions, solid-state spins, photons, and superconducting circuits.^17^

A significant modality of quantum sensing addresses magnetic field sensing, with applications ranging from biomedical to material science. Various techniques have been developed and employed for magnetic field sensing and imaging, e.g. superconducting quantum interference device (SQUID),^18^ magnetic resonance force microscopy,^19^ scanning Hall probe microscopy,^20^ and optical atomic magnetometers^21^ to name a few. These techniques differ in their advantages and disadvantages, offering some combination of high sensitivity and spatial resolution, but sometimes require high vacuum and/or cryogenic temperature to operate.

Quantum sensors based on crystal solid-state defects are promising candidates to circumvent such limitations. NV centers in diamond,^22^ boron-vacancies in hexagonal boron nitride,^23^ silicon-vacancy in diamond and silicon-carbide vacancies^24^ are leading examples of such solid-state defects. Among various solid-state defects, NV centers have been widely explored for room temperature magnetic sensing.^25^ NV centers with long spin coherence time^26^ can be optically initialized, manipulated, and read out under ambient conditions.^27^ Further, the optical transitions of NV centers are highly sensitive to physical conditions such as temperature,^28^ pressure,^29^ electric field^30^ and magnetic field^26^ which makes them well suited for quantum sensing.

NV center based magnetometers demonstrated femto-THz^–1/2^ order magnetic sensitivity.^31^ This technique finds applications in biomedical science^32^ for magnetic imaging of living cells,^33^ magnetic field sensing in artificial magnetic nanoparticles for drug delivery,^34^ monitoring the drug efficacy^35^ and sensing free radical generation in cells.^36^ NV centers were also employed for sensing in condensed matter physics,^37^ e.g. for understanding the charge, spin, and magnetic behavior at the nanoscale in the recently discovered 2D vdW materials^38^ and topological insulators.^39^

### 1.2 Magnetic vdW Materials

The discovery of 2D vdW materials has opened a broad range of novel research fields,^40^ as well as new avenues for applications, such as next-generation computational and spintronic devices,^41^ and has been extensively explored in the past decade. A variety of 2D materials including ferromagnets,^9^ antiferromagnets,^42^ superconductors,^43^ insulators,^44^ and semiconductors^45^ have been studied from cryogenic to room temperature, to understand their magnetic response at the nanoscale with the aim of identifying the different behavior of the 2D material from its original bulk crystal.^37^

One of the vdW magnets of interest is iron–germanium–telluride Fe_(5–x)_GeTe_2_ (FGT), due to its near room temperature T_C_,^13^ making it a suitable candidate for real-world nanospintronic applications. FGT is a cleavable 2D vdW ferromagnet with long-range ferromagnetism originating from significant spin polarization of delocalized ligand Te states.^46^ FGT exhibits a tunable magnetic phase transition between ferromagnetism and antiferromagnetism by applying gate voltage, making it a suitable candidate for fast and energy-efficient data storage devices.^47^ In addition to using 2D magnetic materials for information transfer, spin structures such as magnetic skyrmions, domain-walls, and spin waves can serve as information carriers. In this context, FGT hosts these spin structures at room temperature.^48^

Due to the reduced dimensionality, these 2D magnetic materials have unique properties depending upon their growth conditions and thicknesses compared to their bulk counterparts. In thin layers, the geometric shape factor promotes in-plane magnetic anisotropy and this competes with out-of-plane magnetic anisotropy (or enhances magnetism that intrinsically has a preference for in-plane moment orientation). In some materials, the competing anisotropies lead to the stabilization of magnetic phases in thin flakes (or films) that are not present in bulk crystals.^49,50^

Here, we image and study FGT magnetization under various conditions, including temperature and external magnetic field, with a focus on the effect of the thickness of FGT flakes on their T_C_ and magnetization structures. High-resolution NV center based magnetic imaging reveals domain structures and correlations, specifically relating magnetization with large-scale crystallographic effects. These results, along with the variations identified in the T_C_ of flakes with thicknesses ranging between 15 and 221 nm, provide fundamental insights into the magnetization in these materials, and anisotropy and interfacial effects, with implications on integrated spintronic devices for specific applications, such as magnetic memories and logic.

## 2 Experimental Setup

The NV sensor used is based on a 3 mm × 3 mm × 0.1 mm single crystal electronic grade diamond from Element Six. The diamond was implanted with a 2 × 10^13^ cm^–2^ dose of 10 keV nitrogen (N^15^) ions, followed by annealing and electron irradiation to create an NV center ensemble layer ∼20 nm below the diamond surface. The diamond was cut perpendicular to the crystallographic *x*-axis i.e. along the {1 0 0} plane.^51^ The schematic of a diamond lattice hosting an NV center defect is depicted in Figure 1(a). In this lattice configuration, a bias magnetic field along the *z*-axis has equal projections on all the four possible NV center axes, to be specific B_∥_ = B_z_^ext^·cos­(54.75°), where B_∥_ is the component of B_z_^ext^ parallel to NV center axis.

**Figure 1:**
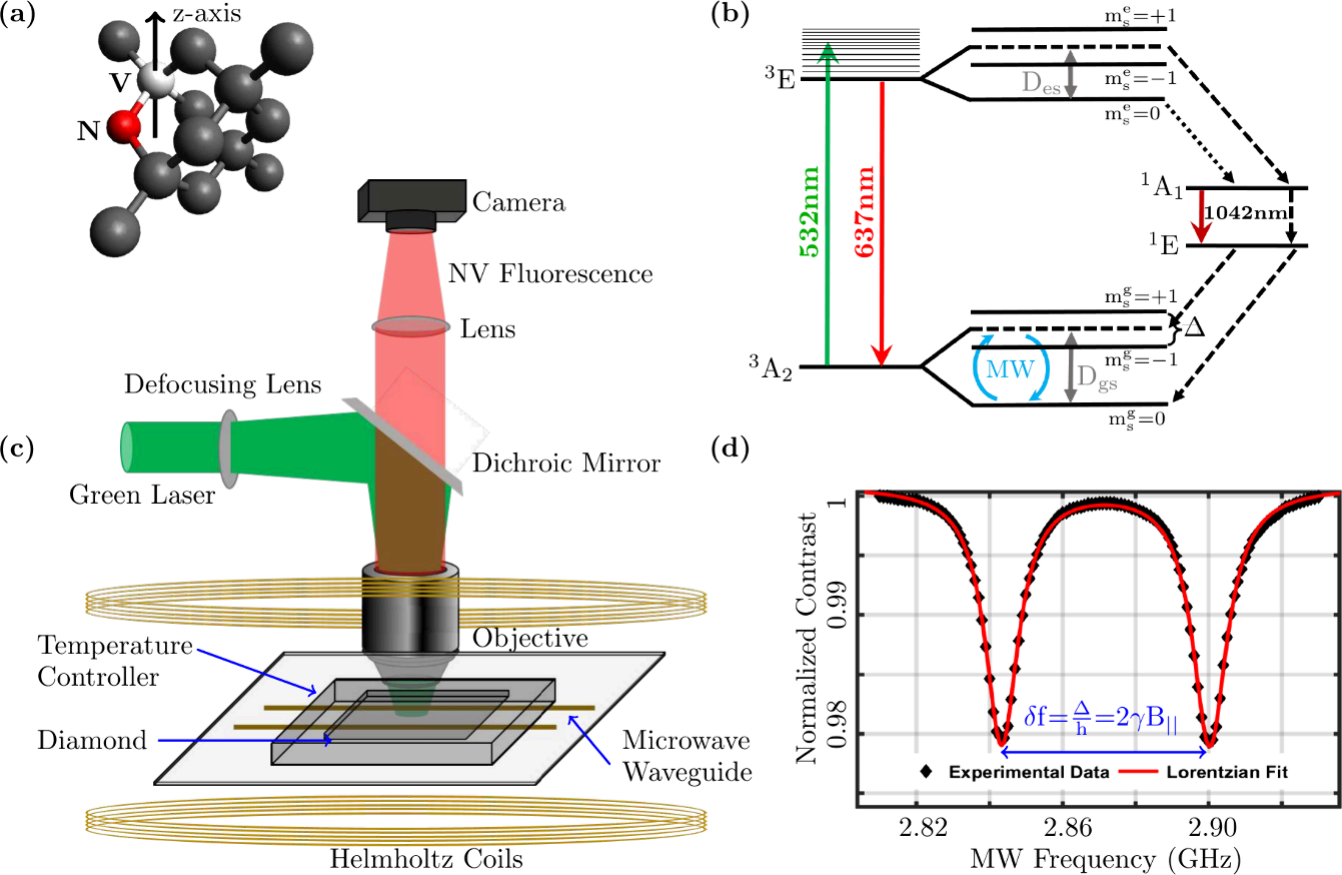
(a) Diamond lattice hosting a nitrogen (red) vacancy (white) defect. Black balls represent carbon atoms. An external bias magnetic field is applied along the *z*-axis. (b) The spin-1 NV center energy level diagram depicting optical pumping from the ground triplet (^3^A_2_) to the excited triplet (^3^E) which may decay via various channels. Dotted/dashed arrows represent optically forbidden transitions. Each spin state is labeled with the respective magnetic spin quantum number, m_*s*_. Δ represents the Zeeman splitting. D_gs_ = 2.87 GHz is the ground state zero-field splitting, excited state zero-field splitting D_es_ = 1.42 GHz, and MW represents the microwave control. (c) Schematic of the experimental setup of NV center QDM. (d) A typical ODMR under an external bias magnetic field. Black diamonds (⧫) are the experimental points while the red line is a Lorentzian fit.

Figure 1(b) depicts a typical energy level diagram of an NV center. A green laser (532 nm) initializes the NV center ensemble in the |m_s_^g^ = 0⟩ or simply |0⟩ state. This is achieved by optically pumping the population of the NV center ensemble from the ground triplet (^3^A_2_) state to the excited triplet (^3^E) states. While the system is in the excited triplet (^3^E) state, it has several decay paths with different probabilities. Optical transitions can take the NV back to the ground triplet (^3^A_2_) and are spin preserving. Non radiative transitions to the intermediate singlet state (^1^A_1_) are shown by dashed/dotted arrows in Figure 1(b). The *m*_*s*_^*e*^ = ± 1 states have a higher decay probability (dashed arrow, Figure 1(b)) than the *m*_*s*_^*e*^ = 0 state (dotted arrow, Figure 1(b)) to the singlet state ^1^A_1_. Hence the spin preserving optical relaxation from the excited triplet (^3^E) to the ground triplet (^3^A_2_) is suppressed for *m*_*s*_ = ±1, which in turn builds up the |0⟩ population. Both radiative and nonradiative decays, from the ^1^A_1_ to the ^1^E singlet, are possible while nonradiative decay from ^1^E singlet to the ^3^A_2_ triplet has identical probabilities for |0⟩ and |±1⟩ (marked by the dashed arrow in Figure 1(b)). An optical transition from the excited triplet state to the ground triplet state gives rise to a characteristic fluorescence at a wavelength of 637 nm i.e. the zero phonon line, with a broad phonon sideband (637–800 nm).

The experimental setup Figure 1(c) includes (in addition to the optics) microwave (MW) delivery, which can induce transitions between the levels |0⟩ and |±1⟩. For the population distribution of the optically pumped equilibrium state, any coherent manipulation between the |0⟩ and |±1⟩ levels using MW drive, results in a decreased fluorescence as depicted in Figure 1(d). In the absence of any external bias magnetic field, one would expect the lowest fluorescence, hence extremum normalized contrast, when the MW drive frequency is swept around D_gs_ = 2.87 GHz, i.e. the zero-field splitting (ZFS) between |0⟩ and |±1⟩.

An external bias field Figure 1(c) in the *z*-direction, typically 30–35 G, is applied using Helmholtz coils to remove the degeneracy of |±1⟩ which results in Zeeman splitting of the NV center ground state manifold by an amount Δ = 2γB_∥_, Figure 1(b), where γ is the electronic gyro-magnetic ratio. We note that B_⊥_, i.e. the component of the external bias magnetic field perpendicular to the NV center axis, competes with the ZFS, and its effect can be neglected on the Zeeman splitting under first-order approximations.^9^ A coplanar MW waveguide is used to coherently control the transitions between the levels |0⟩ and |±1⟩. Experiments were performed at room temperature. To further control the temperature of the sample under investigation, in the range 5°–55 °C (i.e., 278–328 K), a Peltier temperature controller was employed.

Finally, air objectives with 40 and 60 magnifications, having numerical apertures (NA) 0.65 and 0.95 respectively, were used for excitation and collection of the NV center fluorescence. The collected fluorescence was recorded using an Andor Neo 5.5 sCMOS camera, with 2560 × 2160 active pixels (5.5 Megapixels). Depending upon the objective magnification the pixel size, with an appropriate camera binning, is generally of the order of the diffraction limit ≈ λ/2NA ∼ 300 nm. It is worth noting that the experiment is designed such that the camera records the fluorescence pixel-wise, for the duration of the exposure time, at each MW frequency. One can extract the pixel-wise optically detected magnetic resonance (ODMR) for the chosen region of interest (ROI). To compute the contrast at a certain MW frequency one looks for the change in the fluorescence with and without MW drive. This is followed by averaging the ODMR data over all the pixels in the ROI to generate the resulting ODMR plot. A typical ODMR plot under a bias field is shown in Figure 1(d).

## 3 Quantum Magnetic Sensing with NV Centers

An external bias magnetic field removes the degeneracy of the |±1⟩ manifold. The Zeeman splitting between |+1⟩ and |−1⟩ i.e. Δ = 2γB_∥_, can readily be measure experimentally from the ODMR, Figure 1(d), which eventually can be used to compute B_∥_ and hence B_*z*_. In an actual sensing experiment, B_*z*_ could be composed of the applied bias field B_z_^ext^ and the stray field to be sensed. The stray z-field detected by the QDM will be denoted by B_*z*_. As the experimental setup is designed to acquire the pixel-wise ODMR, one may experimentally find the magnetic field seen by each pixel with a spatial resolution limited by the diffraction limit.

### 3.1 Magnetic Sensitivity

To evaluate the magnetic sensitivity of a magnetic sensor, similar to what is employed here, one may typically be interested in computing the signal-to-noise (SNR) limited minimum detectable magnetic field (δB^min^) from an ODMR spectrum.^52^ It can be obtained using the expression

1 $$\delta B_{\min}=\frac{\delta \beta }{\gamma \cdot {\left(\frac{\partial \beta }{\partial \nu }\right)}_{\max}}$$

where δβ is the signal noise at the point of maximum slope $(\frac{\partial \beta }{\partial \nu }{)}_{\max}$ of the ODMR spectrum. The sensitivity, η, for the detection duration *t* is given by η = δ*B*_min_ · √*t*.^52^ The sensitivity, corresponding to the ODMR shown in Figure 1(d), is 172.3 ± 6.8 $\mathrm{nT}/\sqrt{\mathrm{Hz}}.$

### 3.2 FGT Sample

Identifying and synthesizing a 2D vdW magnetic material with room temperature T_C_ is challenging. After the discovery of ferromagnetism in Fe_3_GeTe_2_ with T_*C*_ ∼ 220 K, efforts were made to identify related van der Waals ferromagnets with higher ordering temperatures by employing thicker Fe–Ge slabs into the structure. Such efforts resulted in the discovery of Fe_(5–*x*)_GeTe_2_ materials with ferromagnetism reported near room temperature.^12^ Recent work showed that with suitable doping using cobalt^53,54^ or nickel^55^ one can reach above room temperature T_C_.

The sample used in the current study is a cleavable ferromagnetic 2D vdW crystal Fe_(5–*x*)_GeTe_2_. The crystals were quenched as described in^12^ and directly used without further processing; the experimental value of x is approximately 0.3 based on wavelength dispersive spectroscopy as reported in ref 12. Supporting Information Section-S1 ^56^ details the process for exfoliation and transfer of FGT flakes. Depending upon the iron content ‘x’, the FGT bulk single crystal has a T_C_ ranging from 260 to 310 K^16^ and retains magnetism down to a few nm thickness.^13^ In contrast, depending upon the flake thickness, the T_C_ for 2D flakes ranges between 270 and 300 K.^8,13^ It is interesting to note that the T_C_ not only depends upon the thickness but also on other factors such as the thermal and magnetic history of the bulk material, chemical and mechanical properties of the crystal, etc. Typically, the FGT flakes are observed to have an out-of-plane (OOP) magnetization. The induced magnetization pattern depends upon the bias field strength and temperature.^8,13,57^ This OOP magnetization makes FGT a good candidate to study under only a z-oriented bias field (even though our QDM setup is designed to extract arbitrarily directed magnetization).

### 3.3 Bulk FGT Measurements

To determine the T_C_ of the parent bulk single crystal FGT used in this work, the magnetic moment was measured using a Quantum-Design SQUID magnetometer as a function of temperature under an external magnetic field of 100 Oersted, with zero field cooling (ZFC).

Zero field cooling implies that the FGT bulk sample was cooled to near zero Kelvin under zero magnetic field. Once cooled, a 100 Oersted magnetic field was applied to magnetize the sample and the magnetization was recorded with increasing temperature. The results are depicted in Figure 2, revealing a pair of transitions at around 95 and 133 K which are visible in the zoomed-in plot of Figure 2(b).

**Figure 2:**
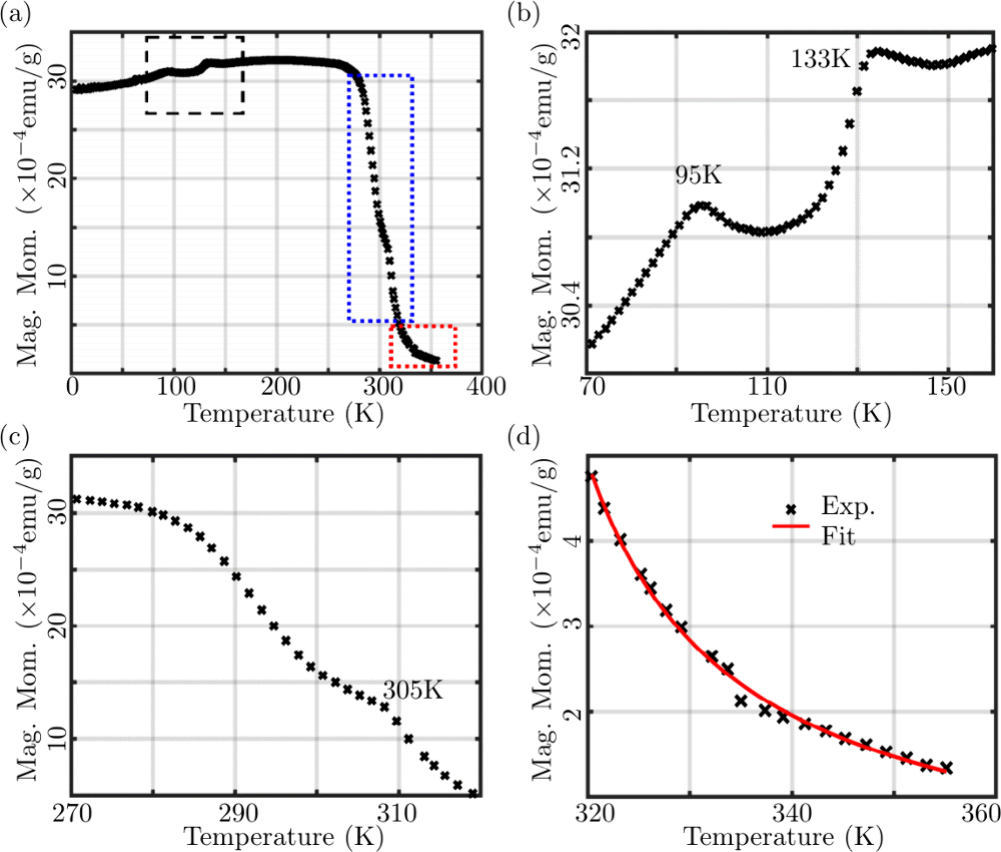
Magnetization characterization of bulk FGT single crystal. (a) Zero field cooled magnetic moment as a function of the temperature under 100 Oersted magnetic field. (b) A zoomed-in plot of the black dashed square in (a) showing two transitions at 95 and 133 K. (c) A zoomed-in plot of the blue dashed rectangle in (a) showing ferromagnetic to paramagnetic phase transition at ∼305 K. (d) A zoomed-in plot of the dotted red rectangle in (a) showing the paramagnetic phase fitted using Curie–Weiss law giving T_C_ = 305.5 ± 2.5 K.

Upon warming in the ZFC condition, the magnetization of the bulk FGT reaches a maximum between 170 and 250 K. At higher temperatures (∼260 K), the magnetization starts decreasing, and as the temperature further increases, the FGT transitions from the ferromagnetic to paramagnetic phase, Figure 2(c). One may observe a kink at ∼305 K displaying the magnetic phase transition of bulk FGT. Above T_C_, the ZFC branch exhibits the typical paramagnetic shape and fitting the data by the Curie–Weiss law (red curve in Figure 2(d)) given by the equation^58,59^

2 $$\chi =\frac{C}{T-\Theta }$$

*χ* being magnetic susceptibility, *C* is the material specific Curie’s constant and Θ is the Weiss constant^59^ typically larger than Curie temperature. This yields Θ = 305.5 ± 2.5 K, a value which fits well with T_C_ mentioned above, Figure 2(c).

## 4 Quantum Magnetic Imaging of FGT Flakes

Under ambient conditions, we study FGT flakes utilizing our in-house developed wide-field NV center based QDM. An optical image at 100× magnification of one such mechanically exfoliated flake, see Supporting Information Section-S1 ^56^ for detail, transferred onto the diamond surface is shown in Figure 3(a). The flake’s average thickness was measured as 137 nm using an atomic force microscope (AFM) (Supporting Information Section-S2 ^56^). The AFM topograph of the FGT flake is shown in Figure 3(b). The extracted stray magnetic field image from the pixel-wise ODMR data is shown in Figure 3(c). Carefully observing Figure 3(a) & (b) reveals stripes which are not noticeable in Figure 3(c) or (d). These stripe features becomes prominent at elevated temperature (see Figure 4) and are explored in detail in Sec.-4.3. The experiment was performed at 288 K and ∼1800 μT z-bias magnetic field. The QDM reveals submicrometer scale magnetic domain features indicating the presence of ferromagnetism and a phase transition expected at room temperature or above for this material. The typical average domain width for the bulk FGT is reported ≈250 nm at 50 K utilizing X-ray photoemission electron microscopy (XPEEM). In addition, the usual domain structure with well-defined domain-walls ceases to exist as the thickness of the FGT flakes decreases. XPEEM-based analysis to visualize the OOP magnetic field for several layers of FGT was reported recently.^60^

**Figure 3:**
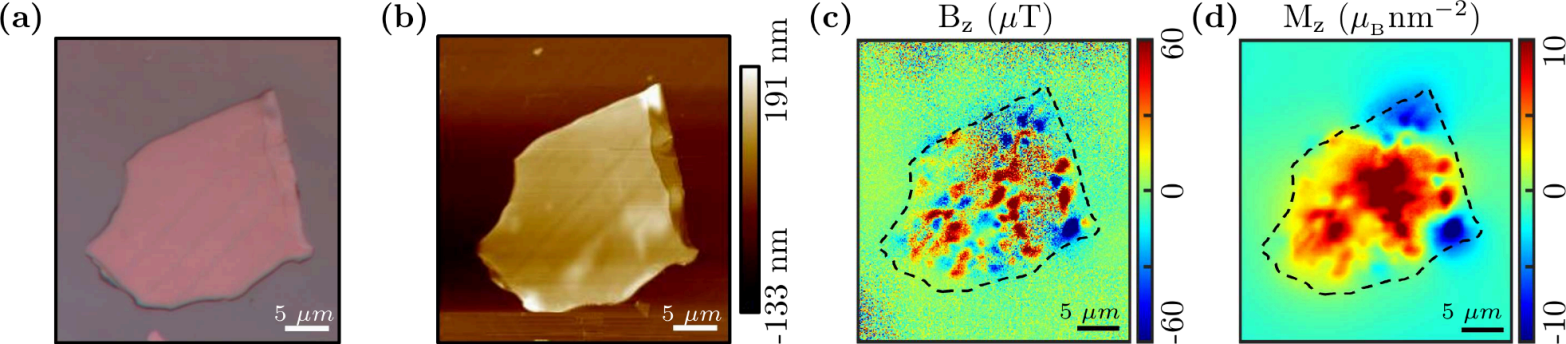
(a) Optical image of an FGT flake transferred onto the diamond surface. (b) AFM topography of the FGT flake. (c) The extracted stray magnetic field, sensed by QDM, of the FGT flake under a 1800 μT z-bias field at 288 K. (d) The reconstructed OOP magnetization (M_*z*_).^9,61^

**Figure 4:**
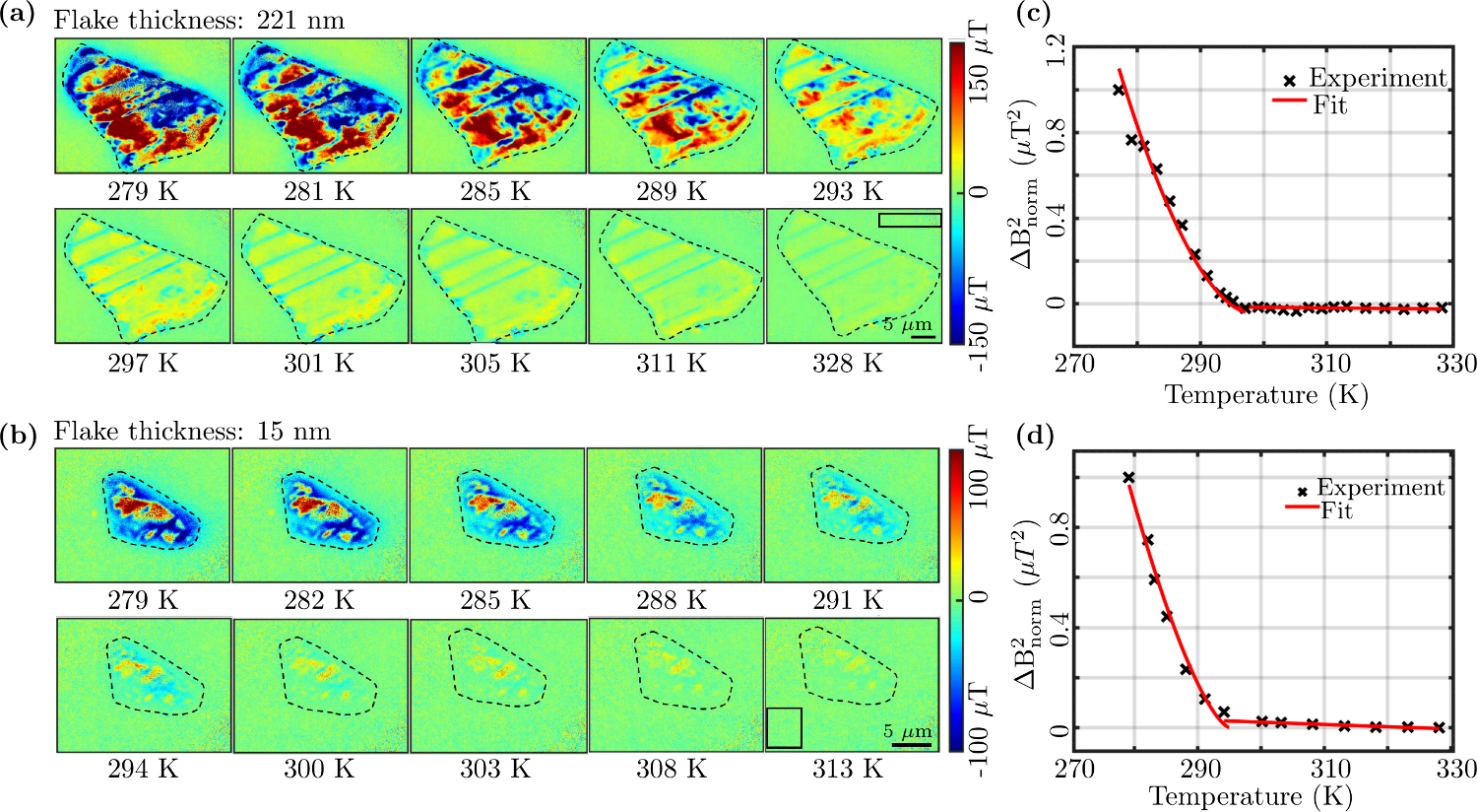
Temperature-dependent QDM images and phase transition plots. (a) Experimentally extracted OOP stray magnetic field (B_*z*_) of 221 nm thick FGT flake under 3150 μT z-bias field for the temperature in range 279–328 K. (b) Experimentally extracted OOP stray magnetic field (B_*z*_) of a 15 nm thin FGT flake under 3150 μT z-bias field for the temperature in range 279–313 K. (c) The stray magnetic field variance ΔB_norm_^2^ versus temperature plot for the FGT flake shown in (a). The retrieved T_C_ is 296 ± 1.5 K. (d) The stray magnetic field variance ΔB_norm_^2^ versus temperature plot for the FGT flake shown in (b). The retrieved T_C_ is 295 ± 2.1 K. Black rectangles in (a) and (b) are the background, and the dashed line encapsulating the flakes are the regions considered for computing the normalized stray field variance ΔB_norm_^2^.

Further, to reconstruct the OOP magnetization (M_*z*_), corresponding to the measured OOP B_*z*_ of the FGT flake shown in Figure 3(c), the method of reverse propagation of magnetization was utilized.^9,61^ The reverse propagation approach allows for the reconstruction of a 2D magnetization map from the measured 2D stray magnetic field. The details of the method used for computing M_*z*_ given the B_*z*_ map of a 2D source, based on the proposals in ref 61, can be found in the Supporting Information of ref 9. Figure 3(d) depicts the reconstructed 2D magnetization image of the FGT flake. Supporting Information Section-S3 ^56^ describes the steps required for this calculation.^9,61^

### 4.1 Temperature Dependence of Stray Fields

For insights into the phase transition and to compute the transition temperature (T_C_), we performed experiments characterizing the temperature dependence of the B_*z*_ of the FGT flakes. A feedback-based Peltier temperature controller, with ± 0.1 K precision, was employed to control the temperature of the flakes (Supporting Information Section-S4 ^56^). In a typical temperature-dependent measurement of B_*z*_, the temperature was varied between 278 and 328 K.

Figure 4 depicts one such temperature-dependent measurement of the stray magnetic fields for two different FGT flakes with 221 nm (thick) and 15 nm (thin) thicknesses. Several representative images of the FGT flake, at different temperatures, are shown here (additional measured magnetic images and extracted magnetization plots are given in the Supporting Information^56^ Section-S6 and S8 respectively). In Figures 4(a) and (b), the temperature-dependent behavior of the B_*z*_ can be observed. One may notice the submicrometer-sized magnetic domains. These domains were observed to preserve their spatial location with time as well as with increase in temperature. At elevated temperatures one may observe the expected diminishing of the stray fields for most of the domains. It is interesting to note domain’s stray field start diminishing while they are locked in their respective positions. Further, near the phase transition domains almost lose their identity and the FGT flake appears mostly like a paramagnet. At lower temperatures, domains have stray fields of the order of 250 μT at a distance of ∼20 nm. With the increase in temperature, domains seem to rearrange and appear to shrink during the phase transition and then completely vanish at elevated temperatures. The phase transition of 2D FGT flakes from ferromagnetic to paramagnetic phase is characterized by a near room-temperature T_C_.^8,13^

For the phase transition plot of the FGT flakes, to extract T_C_, the normalized stray field variance (ΔB_norm_^2^)^8^ is calculated. The ΔB_norm_^2^ versus temperature plot for the thick and thin flakes are shown in Figure 4(c) and (d) respectively. The variance of the flake’s stray field (ΔB_flk_^2^) is calculated from the average B_*z*_ of the flake. The pixel-wise absolute value of B_*z*_ of the flake region, marked by the dashed line in Figures 4(a) and (b), was considered to compute the average B_*z*_ at a particular temperature. The background magnetic field variance (ΔB_bkg_^2^) is calculated from the region enclosed by the black rectangles in Figures 4(a) and (b).

To compute ΔB_norm_^2^, ΔB_bkg_^2^ is subtracted from ΔB_flk_^2^ to remove the background noise followed by appropriate normalization. With an increase in temperature, the vanishing stray-field variance of the flakes indicates the loss of ferromagnetic ordering in 2D FGT flakes as shown in Figures 4(c) and (d), complemented by the respective magnetic images. The experimental data can be fitted by a power law of the form α + β·(1 – T/T_C_)^γ^ ^8,62^ to extract T_C_. From the fitted power law in Figures 4(c) and (d) the values of T_C_ are 296.6 ± 1.5 K and 295 ± 2.1 K, while γ is 1.78 and 1.48, respectively. This temperature-dependent study expands previous work, and indicates the presence of ferromagnetic ordering and near room-temperature transition temperatures in 2D FGT flakes of varied thicknesses, making this material a suitable candidate for practical spintronic devices.

### 4.2 Thickness Dependence of Transition Temperature and Stray Fields

Based on the capabilities of capturing magnetic images of 2D flakes at varied temperatures with our QDM as described above, we studied the thickness-dependence of the phase transition temperature and the stray fields of the FGT flakes. The T_C_, for each flake investigated, is extracted following the procedure explained in Sec.-4.1. Following the methods developed for measuring B_*z*_ and computing T_C_, several FGT flakes with varied thicknesses in the range of 15–221 nm were considered. Figure 5(a) depicts the observed behavior of T_C_ for varied FGT flake thickness. This study shows *no dependence* of T_C_ on the flake thickness in the range of 15–221 nm. These results are consistent with earlier studies, which explored the thickness-dependence of T_C_ for Fe_5_GeTe_2_ in the range 21–100 nm.^14^ Importantly, our work extends previous results and reports a wider thickness range of the FGT flakes. Also, measured several flakes, analyzing T_C_ vs thickness reported as the average thickness of one FGT flake, while earlier studies measured variable thicknesses in a single FGT flake. We note that an anomalous Hall effect-based study of layer number dependent T_C_ ^4^ of Fe_5_GeTe_2_ showed an effect of thickness on T_C_ in a bilayer and a monolayer, where T_C_ falls below room temperature.

**Figure 5:**
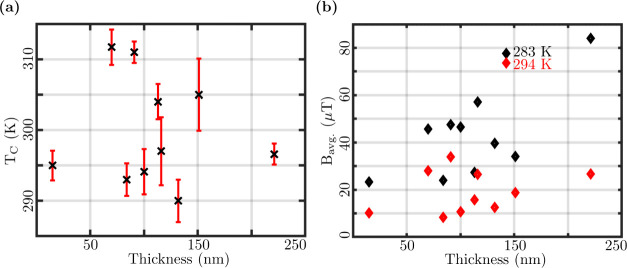
(a) T_C_ versus FGT flake thickness plot. (b) Average magnetic field of an FGT flake as a function of the flake thickness at temperatures 283 K (black diamond) and 294 K (red diamond).

We emphasize that the current study do not contradict the well-established out-of-plane magnetic anisotropy in ferromagnetic van der Waals systems such as FGT. Rather, our measurements indicate that within the investigated thickness range (15–221 nm), the effective magnetic anisotropy remains relatively stable and does not exhibit a pronounced variation with thickness. The observed domain structures–including the characteristic stripe-like features–reflect the presence of strong perpendicular magnetic anisotropy together with interfacial effects, yet their magnitude does not change appreciably across this range. Consistent with this picture, the lack of measurable T_C_ variation between 15 and 221 nm indicates that all examined flakes behave in a bulk-like manner*:* strong interlayer exchange coupling and the three-dimensional Fe network ensure robust long-range ferromagnetic order, while surface*/*interface contributions remain secondary to the dominant magnetocrystalline anisotropy energy. Prior studies have shown that significant T_C_ reduction arises only below ∼5 nm, where weakened interlayer coupling and enhanced surface disorder/oxidation partially suppress magnetic ordering.^4,49^ Current studies support a crossover from two-dimensional to bulk-like ferromagnetism near this threshold thickness; beyond it, T_C_ remains essentially constant, and the interplay between anisotropy, interlayer coupling, and magnetization saturates, yielding bulk-like behavior even in flakes as thin as ∼15 nm.

Moreover, it is worth noting that Fe_5_GeTe_2_ is a ferromagnet, with a near-room temperature T_C_, down to 15 nm thickness. Figure 5(b) shows the B_*z*_ as a function of FGT thickness at 283 and 294 K. Overall, there is an uptrend with the flake thickness. This uptrend was expected as thicker flakes have bigger volume to get magnetize hance produces larger stray fields. The stray fields (at 283 K i.e. below T_C_) as a function of thickness plotted in Figure 5(b) are similar to the results reported earlier.^14^

An extensive study of the domain structure as a function of the number of layers of FGT flakes using XPEEM is reported in ref 60, and T_C_ for a monolayer was found to be in the range 120–150 K. Comparatively, the domain size of poly crystalline Mn_3_Sn films is explored as a function of thickness from a few tens of nanometers to 400 nm, indicating an increase with thickness.^42^

These previous studies showed that the size of the domains critically depends upon the flake thickness, which indeed dictates the overall magnetization of the flake. Earlier results^8,42,60^ were obtained on antiferromagnetic materials, at cryogenic temperatures or cobalt-substituted FGT, while the current study is of FGT flakes at ambient temperature. We find that within the examined range of thicknesses (15–221 nm) interfacial and anisotropy effects are not significant, leading to a thickness-independent T_C_. This is a main result of this work.

### 4.3 Autocorrelation Analysis of Phase Transitions

We analyzed the evolution of magnetic features in FGT flakes and their T_C_ as a function of temperature using a normalized 2D correlation function. This analysis provides deeper insights into T_C_, as well as into the structure of magnetic domains near T_C_, with an external bias magnetic field in magnetic materials.

The normalized 2D autocorrelation function can be written as follows.

3 $$\underset{x,y}{\sum }\frac{B_{\mathrm{stray}}\left(x+\delta x,y+\delta y\right)\cdot B_{\mathrm{stray}}\left(x,y\right)}{∥B_{\mathrm{stray}}\left(x+\delta x,y+\delta y\right)∥\cdot ∥B_{\mathrm{stray}}\left(x,y\right)∥}$$

Here, B_stray_ is the stray magnetic field of an FGT flake at pixel (*x*,*y*) and *δx* and δy are displacements along the *x*-axis and *y*-axis, respectively. The summation quantifies the similarity between the magnetic field at (*x*, *y*) to the field at (x+δx,y+δy). Hence, this function can be used to find the characteristic scale and behavior of magnetic features and textures of a magnetic image.

Figure 6(a) shows temperature-dependent magnetic images of a 100 nm thick FGT flake having T_C_ = 294.1 ± 3.2 K. For all magnetic field images of this flake, refer to Supporting Information Section-S6.^56^ At 276 K, the magnetic domains are relatively large, indicating strong ferromagnetic order. As the temperature increases, the size of the domains starts to shrink, reflecting a gradual weakening of the ferromagnetic state due to thermal agitation. Eventually, as the temperature approaches T_C_, the domains disappear entirely, indicating the transition from a ferromagnetic to a paramagnetic phase. Figure 6(b) shows the corresponding 2D autocorrelation maps at different temperatures, providing a quantitative perspective on the magnetic domain evolution. At 276 K, the autocorrelation map is nearly uniform with a broad full-width at half-maximum (fwhm) around the central peak [(*δx*,*δy*) = (0,0)] indicating large and coherent domains. This broad fwhm corresponds to a high degree of correlation across the image, reflecting strong ferromagnetic ordering.

**Figure 6:**
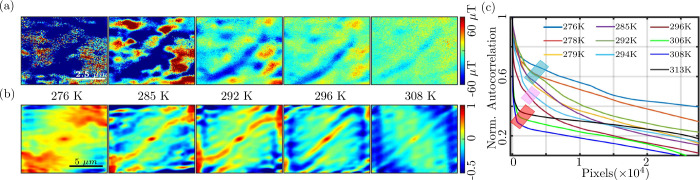
(a) Temperature-dependent evolution of magnetic domains textures in a 100 nm thick FGT flake at several temperatures. (b) Normalized 2D autocorrelation maps, corresponding to the magnetic domains textures shown in (a), show a decrease in the spatial correlation as temperature increases. (c) Autocorrelation function, eq 3, of the magnetic textures are plotted against the number of pixels at several temperatures. At low temperatures, the correlation is high and is highlighted by a blue box. As the temperature increases, the correlation starts decreasing highlighted by a pink box. At higher temperatures above T_C_, the correlation almost vanishes, highlighted by a red box.

As the temperature increases, the fwhm of the autocorrelation peak decreases, indicating a reduction in the spatial extent of correlated regions. This change is visible in the autocorrelation maps, which show areas of positive correlation (red regions) and negative correlation (blue regions) becoming more localized. The shrinking correlation lengths correspond to the shrinking domain size as thermal energy disrupts the ordered magnetic state. Well above T_C_, the autocorrelation nearly vanishes, indicating that the material has transitioned into the paramagnetic phase.

In this state, there is minimal correlation between spins across different regions, and the magnetic domains are no longer discernible. These autocorrelation maps provide valuable insights into the evolution of domain sizes with temperature, allowing one to estimate how the magnetic order diminishes as the system approaches and surpasses its T_C_.

For more quantitative analysis of T_C_ from the 2D autocorrelation maps, we converted the 2D data to 1D by vectorizing the autocorrelation irrespective of position. The resulting vector was then sorted in descending order and plotted against the pixel number for various temperatures, as shown in Figure 6(c). From this plot, we observe that at low temperatures, the autocorrelation values are generally high and closely grouped, as indicated by the blue box. This suggests that at lower temperatures, magnetic domains are highly correlated. With increasing temperatures, near T_C_ the correlation begins to decrease and is reflected in the plot marked by a pink box, and after T_C_ the correlation value is lowest marked by a red box.

### 4.4 The Crystallographic Stripe Feature

A stripe feature in Figure 4(a), which becomes clearer at elevated temperatures, is readily observable. A closer inspection of Figures 3(a) and (b) also reveals similar stripe features in the FGT flakes. The FGT flakes were analyzed using SNVM and AFM to gain further insights into the stripe feature and their subdomain magnetic structure at room temperature.

For SNVM, the resolution exceeds the diffraction limit and is controlled by the stand-off distance of the AFM height feedback, typically less than 100 nm. Being closer to the sample surface allows us to resolve finer details of the FGT stray field with a greater signal magnitude while simultaneously carrying out a correlated AFM scan of the sample topography.

SNVM measurements were carried out on a commercial QZabre QSM system with NV center magnetometry tips obtained from the same supplier. All measurements were performed in an enclosure with temperature regulated at 25 ± 0.05 °C by feedback. The tip was kept in contact with the sample surface through conventional (lateral) amplitude-modulated AFM feedback on the amplitude of the capacitance of a tuning fork that the diamond probe is attached to. A microwave antenna in the vicinity of the tip along with optical access from the above allowed for an ODMR experiment to be performed at each pixel of a magnetometry scan, thereby obtaining the stray field along the axis of the NV center. A vector magnet module situated directly beneath the sample stage allowed for the application of an arbitrary bias field, typically along the axis of the NV center to presplit the ODMR peaks. The position of a particular ODMR peak was tracked along the scan. In the presence of strong field gradients, the peak may be temporarily lost, leading to (partial) fit error lines along the course of the measurement. These error lines were excluded from the measurement over the course of routine data analysis.

Results of SNVM measurements on an FGT flake are depicted in Figure 7. On a closer inspection of Figures 4(a), (b) and Figure 7(a) one may observe a quantitative difference in the extracted stray magnetic fields, under similar bias fields, measured by QDM and SNVM. In order to explore this, further experiments were conducted and results are presented in Supporting Information Section-S7,^56^ indicating consistent measurements. Further, the stripe feature in the stray field shown in Figure 7(a) directly coincides with a faint topographical contrast shown in Figure 7(c), on the order of 5 nm. The same stripe feature can also be observed in the luminosity plot in Figure 7(b). The luminosity plot was obtained from the photon count rate captured by the SNVM and reveals further details closely related to the magnetic image shown in Figure 7(a). The line cut shown in Figure 7(d) shows that the stripe region has a depression of the order of 5 nm.

**Figure 7:**
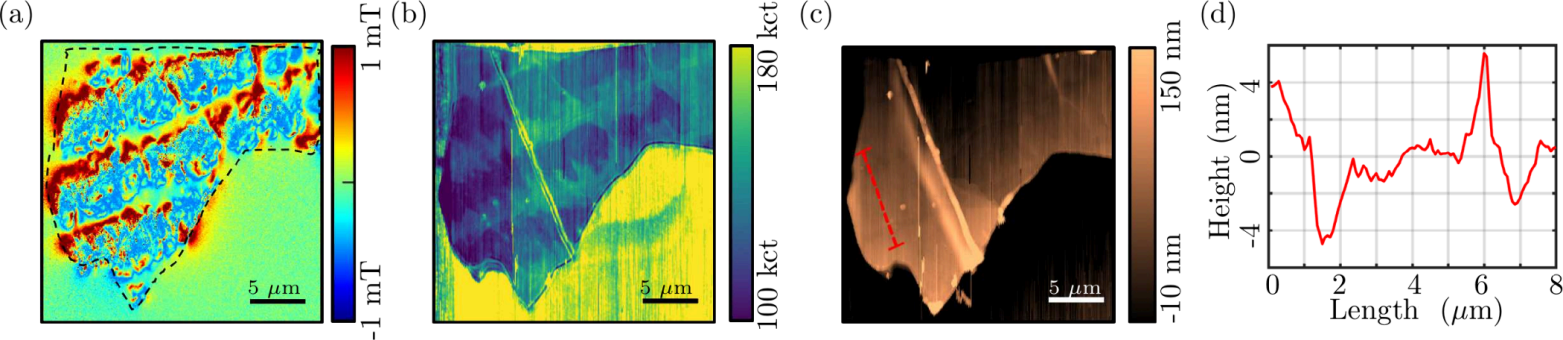
(a) Magnetic image obtained using an SNVM showing the stripe features and domain structure. (b) The luminosity plot depicting the photon count rate captured by SNVM tip. (c) The simultaneous topography, i.e., AFM like, scan captured by the SNVM tip reveals faint signatures of the stripes. (d) A line-cut profile shown by the red dashed line in (c) indicates the stripes with 3–5 nm depression.

For further analysis of the stripe features, energy-dispersive X-ray spectroscopy (EDXS) and AFM measurements were performed. A scanning electron microscope (SEM) image of an FGT flake at 10 keV electron beam energy and 5000 magnification is shown in Figure 8(a). One may observe several stripes. Five keV EDXS measurements were performed on 40 points along a 16.64 μm line as shown in Figure 8(b). Figure 8(c) shows the variations in the atomic percentage for iron, germanium, oxygen, and carbon at the measurement points. Electron beam energy is low for measuring tellurium hence not shown in Figure 8(c). Also, a low energy was chosen for EDXS measurements to examine the near-surface region of the FGT flake. In a higher energy electron beam, we observed tellurium but variations in the atomic percentage for other elements were not as prominent as shown in Figure 8(c).

**Figure 8:**
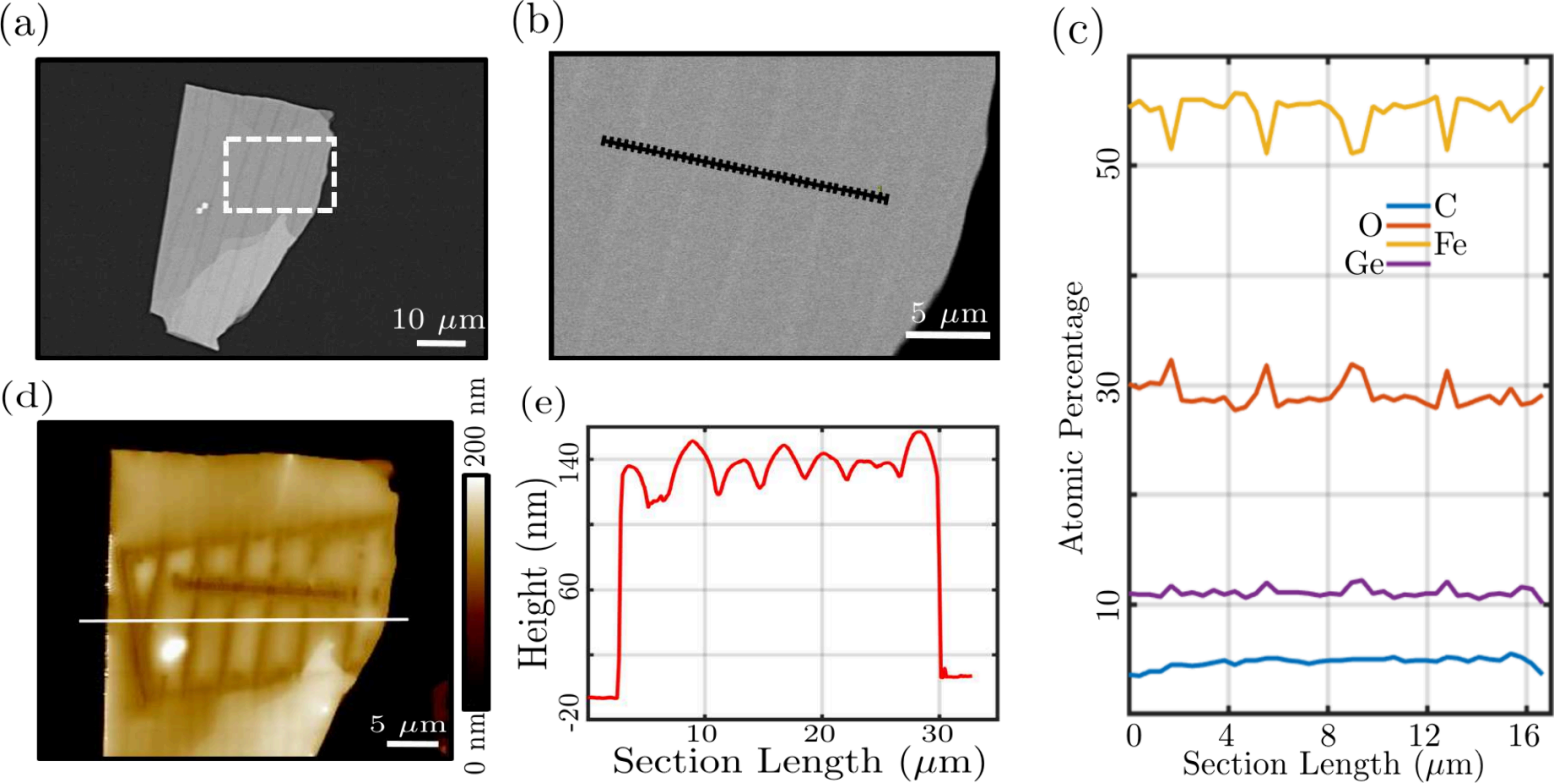
(a) SEM image, at 5000 magnification and 10 keV electron beam energy, of an FGT flake having several stripes. (b) A zoom SEM image of the region enclosed by a white dashed rectangle in (a) at 8000 magnification and 5 keV electron beam energy. The black ladder represents the points considered for the EDXS analysis. (c) EDXS plots, for the region shown by the black ladder in (b), showing the relative atomic percentage of iron, germanium, oxygen, and carbon. (d) The AFM topography of the FGT flake depicting the height profile of the FGT flake. Regions with additional depressions are the results of the exposure to electron beam during SEM imaging. One may observe damage in the region of EDXS analysis. (e) The line profile of the FGT flake is marked by a white line in (d).

One may observe the correlation between the change in concentration of oxygen, iron, and germanium and the respective points on the stripes. These results suggest that there are modulations of iron and germanium concentration in the stripe region. These modulations might be attributed to the respective elemental concentration variations during the synthesis of the FGT. Such modulations caused the surface to oxidize differently compared to the rest of the FGT flake which is further confirmed by the oxygen content on these stripes.

SEM and EDXS measurements were followed by AFM analysis and the obtained topography is shown in Figure 8(d). The e-beam exposed region during EDXS measurement is visible in the AFM image, showing a distinct contrast between the striped regions and the rest of the flake, indicating different interactions of the e-beam with these regions. This could be attributed to the modulation of oxygen, iron, and germanium content in the stripe regions as depicted by the EDXS analysis in Figure 8(c). The line profile in Figure 8(e), along the white line in Figure 8(d), highlights the variation in height across the stripes. Here the depressions are of the order of 10 nm, which seems to be enhanced by the electron beam exposure as compared to the AFM measurement of SNVM Figure 7(d).

It is worth noting that several flakes were examined for the stripe features. The stripe features appears in the magnetic images which were obtained from the surface of the FGT flakes facing the diamond substrate. FGT flakes being encapsulated with gold inside a glovebox were not exposed to oxygen (see Supporting Information Section-S5 ^56^ detailing the effect of oxidation on magnetic image), yet they still exhibit the stripe features in both magnetic and optical images. Hence along with the EDXS and SEM analysis, we conclude that modulation in iron and germanium content during the FGT synthesis, with possibly oxygen also being present, let these stripes oxidize differently leading to our measured results. This might also explain the different FGT behavior in these stripe regions when exposed to a 5–10 keV electron beam, Figure 8(d).

Further analysis of these stripe features was carried out by computing the autocorrelation given by eq 3. Figure 9(a) is an optical image of a ∼130 nm thick FGT flake. The image shows faint stripe features at 100 magnification. These features are also visible in the magnetic image, Figure 9(b), taken at 307 K of the FGT flake. Figure 9(c) is a normalized 2D autocorrelation map of the region marked with a dashed square in Figure 9(b). The same stripe features are also reflected in the autocorrelation map. This map provides a quantitative analysis of the spatial correlation between the stripes, highlighting the periodicity and micron-order size of these magnetic features in the FGT flake. To further analyze these structures, we extracted angle-dependent 1D autocorrelation data from the 2D autocorrelation map in Figure 9(c). The stripes are fairly consistent in spacing, so the average gap between the stripes is plotted as a function of the angle in Figure 9(d). This plot shows that the gap increases with angle and at ∼60 degrees, no gap is detected as around this angle the line along which we are extracting the data becomes parallel to these highly correlated line region. Hence, these features are at ∼60 degree angle w.r.t. the horizontal axis with an average gap ranging between 2 and 3 μm.

**Figure 9:**
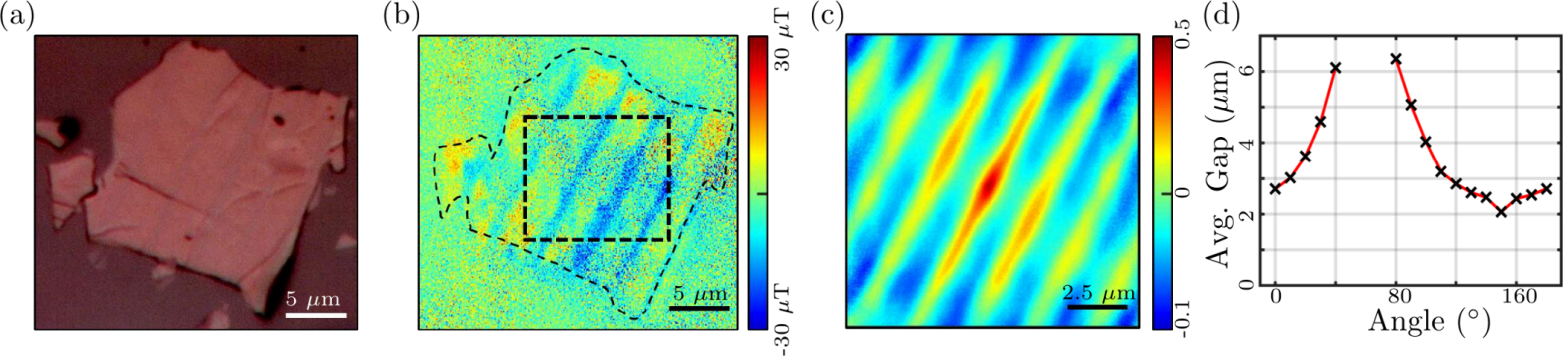
(a) Optical image of an FGT flake at 100 magnification showing stripe features. (b) The magnetic stray field-based image of the FGT flake shown in (a) at 307 K. (c) The normalized 2D autocorrelation map of a region marked with a dotted square in (b). (d) Directional 1D autocorrelation analysis of the stripe features from (c), depicting the angular dependence of spatial correlations.

This unexpected crystallographic feature was not previously identified, and our measurements correlate these micron-scale structures to optical, magnetic and elemental signatures. We deduce that these growth features lead to surface oxidation modifications, and as a result to magnetic implications as well. Importantly, while we have shown interfacial effects are not significant in terms of the average T_C_ of the flakes, such effects, when associated with intrinsic crystallographic features, can lead to local changes in the magnetization distribution. This is another main result of this paper.

## 5 Conclusion

Here, we have demonstrated the capabilities of an NV center based QDM for noninvasive, high-resolution probing of magnetic features in 2D vdW ferromagnets. The NV center magnetometry technique has allowed us to achieve detailed magnetic field imaging at room temperature and under ambient conditions, overcoming limitations of traditional approaches that commonly require high-vacuum and/or cryogenic environments.

The QDM imaging of a cleaved 2D FGT flake revealed submicrometer scale magnetic domains and confirmed room-temperature ferromagnetism in the sample. Temperature-dependent measurements showed clear evidence of phase transitions in the FGT 2D flakes at room temperature, with T_C_ ranging between 285 to 315 K for thicknesses between 15 and 221 nm. This study identified no clear thickness dependence of the T_C_ behavior in Fe_5_GeTe_2_ for the studied thickness range.^4,8,14^ We also measured the T_C_ of a bulk single crystal for comparison with exfoliated 2D flakes T_C_, yet observed no significant variation. Importantly, these results indicate no significant interfacial and anisotropic magnetism effects. We note, however, that our conclusions do not extend to the ultrathin limit (<10 nm), where prior work has shown that bilayer and monolayer^4,60^ FGT exhibit a pronounced suppression of T_C_. Thus, the present results establish the robustness of ferromagnetism in thicker FGT flakes, while emphasizing that distinct physics emerges as the dimensionality is further reduced. Further, the B_*z*_ of FGT flake as a function of flake thickness is presented at low (283 K) and high (294 K) temperatures. This study confirms the presence of ferromagnetism in FGT down to 15 nm, making it a suitable candidate for fabricating high-quality 2D magnetic devices at room temperature for spintronic applications.

We observed a magnetic stripe feature repeatedly appearing during the experiments. It was explored using SNVM, SEM, EDXS, and AFM. We observed that the stripe features can be attributed to modulation in iron, oxygen, and germanium concentrations, which potentially influence surface oxidation, thus leading to these stripes structures. Variations in the oxidation levels were further confirmed by EDXS measurements. We note that these stripe features are not a general feature of FGT flakes but likely result from growth variations, as we did not observe these features in all the available samples. Nevertheless, this structure, which was not previously identified, indicates implications of interface effects on measurable magnetization structures, when accompanied by crystallographic variations.

Moreover, we use high-resolution magnetic imaging and 2D correlation analysis to characterize the magnetic domain structures as a function of temperature. We identify the magnetic spatial behavior across T_C_ through characteristics of the central lobe of the autocorrelation map. Finally, we identify stripe features in these flakes on the micron scale, which manifest through the magnetic signatures and their 2D correlation features. We thus demonstrate the usefulness of such autocorrelation analysis schemes for characterizing spatial magnetization structures.

As future prospects, using an NV center based QDM, one can study current-induced domain-wall motion (CIDM), spin–orbit torque, ferromagnetic resonance, spin-transfer torque, magnetic noises, and excitations. Such quantum sensing schemes are specifically well-suited for studying 2D vdW materials,^15,37,63^ to further understand their underlying physics and to evaluate their potential use in spintronic devices and applications.

## Supplementary Material



## Notes

Information on sample preparation, flake thickness measurement, magnetization extraction, temperature control, thin flake chemical degradation, temperature dependent magnetic imaging, wide-field versus scanning NV spectrometer magnetic imaging, temperature dependent magnetization and the EDXS analysis of FGT flakes^56^ (PDF)

## References

[1] Liu Y., Weiss N. O., Duan X., Cheng H.-C., Huang Y., Duan X. (2016). Van der Waals heterostructures and devices. Nat. Rev. Mater..

[2] Frisenda R., Niu Y., Gant P., Muñoz M., Castellanos-Gomez A. (2020). Naturally
occurring van der Waals materials. npj 2D Mater.
Appl..

[3] Li H., Ruan S., Zeng Y.-J. (2019). Intrinsic
van der Waals magnetic
materials from bulk to the 2D limit: New Frontiers of Spintronics. Adv. Mater..

[4] Deng Y., Xiang Z., Lei B., Zhu K., Mu H., Zhuo W., Hua X., Wang M., Wang Z., Wang G., Tian M., Chen X. (2022). Layer-number-dependent
magnetism and anomalous Hall effect in van der Waals ferromagnet Fe_5_GeTe_2_. Nano Lett..

[5] Meng L. (2021). Anomalous thickness
dependence of Curie temperature in air-stable
two-dimensional ferromagnetic 1T-CrTe_2_ grown by chemical
vapor deposition. Nat. Commun..

[6] Huang B., Clark G., Navarro-Moratalla E., Klein D. R., Cheng R., Seyler K. L., Zhong D., Schmidgall E., McGuire M. A., Cobden D. H., Yao W., Xiao D., Jarillo-Herrero P., Xu X. (2017). Layer-dependent ferromagnetism
in
a van der Waals crystal down to the monolayer limit. Nature.

[7] Klein D. R., MacNeill D., Lado J. L., Soriano D., Navarro-Moratalla E., Watanabe K., Taniguchi T., Manni S., Canfield P., Fernández-Rossier J., Jarillo-Herrero P. (2018). Probing magnetism
in 2D van der Waals crystalline insulators via electron tunneling. Science.

[8] Chen H. (2023). Above-room-temperature ferromagnetism in thin van der Waals flakes
of cobalt-substituted Fe_5_GeTe_2_. ACS Appl. Mater. Interfaces.

[9] Thiel L., Wang Z., Tschudin M. A., Rohner D., Gutiérrez-Lezama I., Ubrig N., Gibertini M., Giannini E., Morpurgo A. F., Maletinsky P. (2019). Probing magnetism
in 2D materials at the nanoscale
with single-spin microscopy. Science.

[10] Yi J., Zhuang H., Zou Q., Wu Z., Cao G., Tang S., Calder S. A., Kent P. R. C., Mandrus D., Gai Z. (2016). Competing antiferromagnetism in a
quasi-2D itinerant ferromagnet:
Fe_3_GeTe_2_. 2D Mater..

[11] Broadway D. A., Scholten S. C., Tan C., Dontschuk N., Lillie S. E., Johnson B. C., Zheng G., Wang Z., Oganov A. R., Tian S., Li C., Lei H., Wang L., Hollenberg L. C. L., Tetienne J.-P. (2020). Imaging domain reversal
in an ultrathin van der Waals ferromagnet. Adv.
Mater..

[12] Stahl J., Shlaen E., Johrendt D. (2018). The van der
Waals ferromagnets Fe_5–*δ*
_GeTe_2_ and Fe_5–*δ*–_xNi_
*x*
_GeTe_2_ - crystal structure,
stacking faults, and
magnetic properties. Z. Anorg. Allg. Chem..

[13] May A. F., Ovchinnikov D., Zheng Q., Hermann R., Calder S., Huang B., Fei Z., Liu Y., Xu X., McGuire M. A. (2019). Ferromagnetism near
room temperature in the cleavable
van der Waals crystal Fe_5_GeTe_2_. ACS Nano.

[14] Chen H., Asif S., Whalen M., Támara-Isaza J., Luetke B., Wang Y., Wang X., Ayako M., Lamsal S., May A. F., McGuire M. A., Chakraborty C., Xiao J. Q., Ku M. J. H. (2022). Revealing room temperature ferromagnetism
in exfoliated Fe_5_GeTe_2_ flakes with quantum magnetic
imaging. 2D Mater..

[15] Robertson I. O., Tan C., Scholten S. C., Healey A. J., Abrahams G. J., Zheng G., Manchon A., Wang L., Tetienne J.-P. (2022). Imaging current
control of magnetization in Fe_3_GeTe_2_ with a
widefield nitrogen-vacancy microscope. 2D Mater..

[16] Alahmed L. (2021). Magnetism and spin dynamics
in room-temperature van der Waals magnet
Fe_5_GeTe_2_. 2D Mater..

[17] Degen C. L., Reinhard F., Cappellaro P. (2017). Quantum sensing. Rev. Mod. Phys..

[18] Greenberg Y. S. (1998). Application
of superconducting quantum interference devices to nuclear magnetic
resonance. Rev. Mod. Phys..

[19] Rugar D., Budakian R., Mamin H. J., Chui B. W. (2004). Single spin detection
by magnetic resonance force microscopy. Nature.

[20] Chang A. M., Hallen H. D., Harriott L., Hess H. F., Kao H. L., Kwo J., Miller R. E., Wolfe R., van der Ziel J., Chang T. Y. (1992). Scanning Hall probe
microscopy. Appl. Phys. Lett..

[21] Ledbetter M. P., Savukov I. M., Budker D., Shah V., Knappe S., Kitching J., Michalak D. J., Xu S., Pines A. (2008). Zero-field
remote detection of NMR with a microfabricated atomic magnetometer. Proc. Natl. Acad. Sci. U. S. A..

[22] Dyer H. B., Raal F. A., Du Preez L., Loubser J. H. N. (1965). Optical absorption
features associated with paramagnetic nitrogen in diamond. Philos. Mag..

[23] Tran T. T., Bray K., Ford M. J., Toth M., Aharonovich I. (2016). Quantum emission
from hexagonal boron nitride monolayers. Nat.
Nanotechnol..

[24] Nagy R. (2019). High-fidelity spin and optical control of single silicon-vacancy
centres in silicon carbide. Nat. Commun..

[25] Barry J. F., Schloss J. M., Bauch E., Turner M. J., Hart C. A., Pham L. M., Walsworth R. L. (2020). Sensitivity
optimization for NV-diamond
magnetometry. Rev. Mod. Phys..

[26] Bar-Gill N., Pham L. M., Jarmola A., Budker D., Walsworth R. L. (2013). Solid-state
electronic spin coherence time approaching one second. Nat. Commun..

[27] Hanson R., Gywat O., Awschalom D. D. (2006). Room-temperature
manipulation and
decoherence of a single spin in diamond. Phys.
Rev. B.

[28] Choe S., Yoon J., Lee M., Oh J., Lee D., Kang H., Lee C.-H., Lee D. (2018). Precise temperature
sensing with nanoscale thermal sensors based on diamond NV centers. Curr. Appl. Phys..

[29] Hilberer A., Toraille L., Dailledouze C., Adam M.-P., Hanlon L., Weck G., Schmidt M., Loubeyre P., Roch J.-F. (2023). Enabling
quantum sensing under extreme pressure: Nitrogen-vacancy magnetometry
up to 130 GPa. Phys. Rev. B.

[30] Dolde F., Fedder H., Doherty M. W., Nöbauer T., Rempp F., Balasubramanian G., Wolf T., Reinhard F., Hollenberg L. C. L., Jelezko F., Wrachtrup J. (2011). Electric-field
sensing using single diamond spins. Nat. Phys..

[31] Xie Y., Yu H., Zhu Y., Qin X., Rong X., Duan C.-K., Du J. (2021). A hybrid magnetometer
towards femtotesla sensitivity under ambient
conditions. Sci. Bull..

[32] Aslam N., Zhou H., Urbach E. K., Turner M. J., Walsworth R. L., Lukin M. D., Park H. (2023). Quantum sensors
for biomedical applications. Nat. Rev. Phys..

[33] Le
Sage D., Arai K., Glenn D. R., DeVience S. J., Pham L. M., Rahn-Lee L., Lukin M. D., Yacoby A., Komeili A., Walsworth R. L. (2013). Optical magnetic imaging of living cells. Nature.

[34] Veiseh O., Gunn J. W., Zhang M. (2010). Design and fabrication of magnetic
nanoparticles for targeted drug delivery and imaging. Adv. Drug Delivery Rev..

[35] Elías-Llumbet A., Tian Y., Reyes-San-Martin C., Reina-Mahecha A., Damle V., Morita A., van der
Veen H. C., Sharma P. K., Sandovici M., Mzyk A., Schirhagl R. (2023). Quantum sensing
for real-time monitoring of drug efficacy in synovial fluid from arthritis
patients. Nano Lett..

[36] Fan S., Lopez Llorens L., Perona Martinez F. P., Schirhagl R. (2024). Quantum sensing
of free radical generation in mitochondria of human keratinocytes
during UVB exposure. ACS Sens..

[37] Casola F., van der Sar T., Yacoby A. (2018). Probing condensed matter physics
with magnetometry based on nitrogen-vacancy centres in diamond. Nat. Rev. Mater..

[38] Geim A. K., Grigorieva I. V. (2013). Van der Waals heterostructures. Nature.

[39] Hasan M. Z., Kane C. L. (2010). Colloquium: Topological insulators. Rev. Mod. Phys..

[40] Novoselov K. S., Mishchenko A., Carvalho A., Castro Neto A. H. (2016). 2D materials
and van der Waals heterostructures. Science.

[41] Ahn E. C. (2020). 2D materials
for spintronic devices. npj 2D Mater. Appl..

[42] Li S., Huang M., Lu H., McLaughlin N. J., Xiao Y., Zhou J., Fullerton E. E., Chen H., Wang H., Du C. R. (2023). Nanoscale magnetic
domains in polycrystalline Mn_3_Sn films imaged by a scanning
single-spin magnetometer. Nano Lett..

[43] Xi X., Wang Z., Zhao W., Park J.-H., Law K. T., Berger H., Forró L., Shan J., Mak K. F. (2016). Ising pairing
in superconducting NbSe_2_ atomiclayers. Nat. Phys..

[44] McLaughlin N. J., Kalcheim Y., Suceava A., Wang H., Schuller I. K., Du C. R. (2021). Quantum
sensing of insulator-to-metal transitions in a Mott insulator. Adv. Quantum Technol..

[45] Manzeli S., Ovchinnikov D., Pasquier D., Yazyev O. V., Kis A. (2017). 2D transition
metal dichalcogenides. Nat. Rev. Mater..

[46] Yamagami K. (2021). Itinerant ferromagnetism
mediated by giant spin polarization of the
metallic ligand band in the van der Waals magnet Fe_5_GeTe_2_. Phys. Rev. B.

[47] Tan C., Xie W.-Q., Zheng G., Aloufi N., Albarakati S., Algarni M., Li J., Partridge J., Culcer D., Wang X., Yi J. B., Tian M., Xiong Y., Zhao Y.-J., Wang L. (2021). Gate-controlled magnetic
phase transition in a van der Waals magnet Fe_5_GeTe_2_. Nano Lett..

[48] Schmitt M., Denneulin T., Kovács A., Saunderson T. G., Rüßmann P., Shahee A., Scholz T., Tavabi A. H., Gradhand M., Mavropoulos P., Lotsch B. V., Dunin-Borkowski R. E., Mokrousov Y., Blügel S., Kläui M. (2022). Skyrmionic
spin structures in layered Fe_5_GeTe_2_ up to room
temperature. Commu. Phys..

[49] Fei Z., Huang B., Malinowski P., Wang W., Song T., Sanchez J., Yao W., Xiao D., Zhu X., May A. F., Wu W., Cobden D. H., Chu J.-H., Xu X. (2018). Two-dimensional itinerant ferromagnetism in atomically thin Fe_3_GeTe_2_. Nat. Mater..

[50] Huang F., Kief M. T., Mankey G. J., Willis R. F. (1994). Magnetism in the
few-monolayers limit: A surface magneto-optic Kerr-effect study of
the magnetic behavior of ultrathin films of Co, Ni, and Co-Ni alloys
on Cu(100) and Cu(111). Phys. Rev. B.

[51] Kittel, C. Introduction to Solid State Physics, 8th ed.; Wiley, 2004.

[52] Dréau A., Lesik M., Rondin L., Spinicelli P., Arcizet O., Roch J. F., Jacques V. (2011). Avoiding power broadening
in optically detected magnetic resonance of single NV defects for
enhanced dc magnetic field sensitivity. Phys.
Rev. B.

[53] May A. F., Du M.-H., Cooper V. R., McGuire M. A. (2020). Tuning magnetic
order in the van der Waals metal Fe_5_GeTe_2_ by
cobalt substitution. Phys. Rev. Mater..

[54] Tian C., Pan F., Xu S., Ai K., Xia T., Cheng P. (2020). Tunable magnetic
properties in van der Waals crystals (Fe_1–*x*
_Co_
*x*
_)_5_GeTe_2_. Appl. Phys. Lett..

[55] Chen X., Shao Y.-T., Chen R., Susarla S., Hogan T., He Y., Zhang H., Wang S., Yao J., Ercius P., Muller D. A., Ramesh R., Birgeneau R. J. (2022). Pervasive
beyond room-temperature ferromagnetism in a doped van der Waals magnet. Phys. Rev. Lett..

[56] Bindu; Singh, A. ; Hen, A. ; Ćavar, L. D. ; Ulrich Schultheis, S. M. ; Yochelis, S. ; Paltiel, Y. ; May, A. F. ; Wittmann, A. ; Kläui, M. ; Budker, D. ; Steinberg, H. ; Bar-Gill, N. Supporting Information: Quantum Imaging of Ferromagnetic van der Waals Magnetic Domain Structures at Ambient Conditions. https://pubs.acs.org/doi/10.1021/acsami.5c16352, 2025. Available as supporting material to the article published in ACS Applied Materials & Interfaces. 10.1021/acsami.5c16352 PMC12635976 41197977

[57] Nava
Antonio G., Bertelli I., Simon B. G., Medapalli R., Afanasiev D., van der Sar T. (2021). Magnetic imaging and statistical
analysis of the metamagnetic phase transition of FeRh with electron
spins in diamond. J. Appl. Phys..

[58] Danielian A. (1962). On interpreting
high temperature magnetic susceptibility data. Proc. of the Phys. Soc..

[59] Rhodes P., Wohlfarth E. P. (1963). The effective Curie-Weiss constant of ferromagnetic
metals and alloys. Proc. of the R. Soc. of Lon.
Ser. A, Math. and Phys. Sc..

[60] Fujita R., Bassirian P., Li Z., Guo Y., Mawass M. A., Kronast F., van der Laan G., Hesjedal T. (2022). Layer-dependent magnetic
domains in atomically thin Fe_5_GeTe_2_. ACS Nano.

[61] Tan S., Ma Y. P., Thomas I., Wikswo J. (1996). Reconstruction of two-dimensional
magnetization and susceptibility distributions from the magnetic field
of soft magnetic materials. IEEE Trans. Magn..

[62] Zhang H., Chen R., Zhai K., Chen X., Caretta L., Huang X., Chopdekar R. V., Cao J., Sun J., Yao J., Birgeneau R., Ramesh R. (2020). Itinerant ferromagnetism in van der
Waals Fe_5–*x*
_GeTe_2_ crystals
above room temperature. Phys. Rev. B.

[63] Johansen O., Risinggård V., Sudbø A., Linder J., Brataas A. (2019). Current control
of magnetism in two-dimensional Fe_3_GeTe_2_. Phys. Rev. Lett..

