# Supplementary material for: Quantum Imaging of Ferromagnetic van der Waals Magnetic Domain Structures at Ambient Conditions
Source: ACS Appl Mater Interfaces. 2025 Nov 6;17(46):63956–67. doi: 10.1021/acsami.5c16352 (PMC12635976; doi:10.1021/acsami.5c16352)
Supplement: Supplementary file 1 [file am5c16352_si_001.pdf]

1

Supporting Information: Quantum Imaging of Ferromagnetic van der Waals Magnetic Domain Structures at Ambient Conditions

Bindu,[a,†] Amandeep Singh[a,*,†] Amir Hen,[†] Lukas Drago Ćavar,[‡] Sebastian Maria Ulrich Schultheis,[‡] Shira Yochelis,[†] Yossi Paltiel,[†] Andrew F. May,[¶] Angela Wittmann,[‡] Mathias Kläui,[‡] Dmitry Budker,[§,‖,#] Hadar Steinberg,[⊥,@] and Nir Bar-Gill[*,†,⊥]

†*Institute of Applied Physics, The Hebrew University of Jerusalem, Jerusalem, 9190401, Israel*

‡*Institute of Physics, Johannes Gutenberg University Mainz, 55128 Mainz, Germany*

¶*Materials Science and Technology Division, Oak Ridge National Laboratory, Oak Ridge, TN 37831, USA*

§*Johannes Gutenberg-Universität Mainz, 55128 Mainz, Germany*

‖*Helmholtz-Institut Mainz, GSI Helmholtzzentrum für Schwerionenforschung, 55128 Mainz, Germany*

⊥*The Racah Institute of Physics, The Hebrew University of Jerusalem, Jerusalem 9190401, Israel*

#*Department of Physics, University of California, Berkeley, CA 94720, USA*

@*The Center of Nano-Science and Nanotechnology, The Hebrew University of Jerusalem, Jerusalem, 9190401, Israel*

E-mail: www.amansidhu.com@gmail.com; bargill@phys.huji.ac.il

[a]These authors contributed equally to this work.

S1. Sample Preparation

Fe_5GeTe_2 (FGT) bulk crystal was kept inside the Argon-filled glove box (<5 ppm of H_2O and <5 ppm of O_2) to prevent oxidation. The thin flakes were mechanically exfoliated from the bulk crystal using silicone-free adhesive plastic tape (Utron Systems Inc., P/N: 1007R-6.0) inside the glove box Fig.S1(a). The sample was repeatedly exfoliated on the tape to get thinner flakes by adhering it to itself Fig.S1(b). These thin flakes were transferred onto the diamond by stamping the tape on the diamond surface Fig.S1(c), with proximity to NV centers. An optical image of transferred FGT flakes onto the diamond surface is shown in Fig.S1(d).

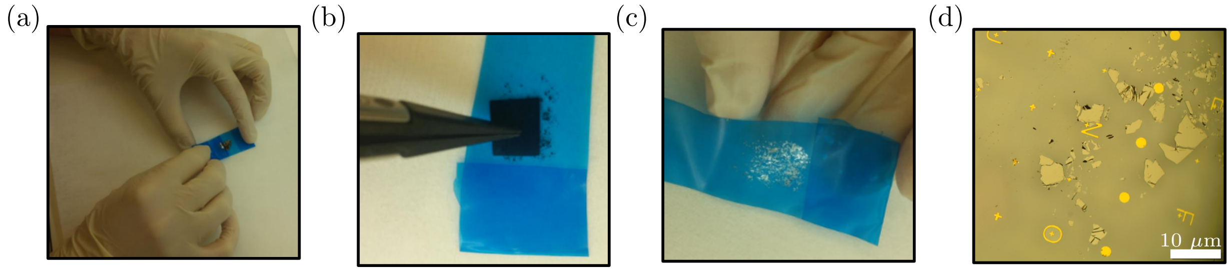

Figure S1: (a) Exfoliation of Fe_5GeTe_2 flakes from the bulk crystal. (b) Thinning the thick flakes transferred from the bulk crystal onto the tape by repeatedly adhering the tape to itself. (c) Stamping the tape for transferring the flakes onto the diamond surface. (d) Optical image of the transferred flakes on the diamond surface having gold markers. Gold markers assist locating the FGT flakes in the wide-field setup.

Gold markers were fabricated on the diamond substrate Fig.S1(d) to locate the flakes in the wide-field setup. 10 nm chromium (Cr) was evaporated on the diamond surface as the adhesive layer followed by a 100 nm gold (Au) layer. The markers were printed on these deposited layers by photolithography using a laser writer (LW405B, Microtech, Palermo Italy). This was followed by etching away the unwanted Au and Cr. The diamond was glued to a silicon chip, Fig.S1(b), for easy handling of the diamond during the flake transfer process.

S2. Flake Thickness Measurement

The thickness of the FGT flakes was characterized using Bruker's dimension XR atomic force microscope (AFM) in tapping mode. The analysis was carried out using Nanoscope analysis software provided by Bruker. Fig.S2(a) depicts an AFM topography, of a FGT flake transferred on the diamond surface. The average thickness is extracted using the step function in Nanoscope analysis software. The step function draws an averaging box with a reference line in the center S2(a) and generates a height profile from the averaged data from both sides of the reference line in the box S2(b). By adjusting the cursor on the height profile average thickness is measured. The average thickness of this particular flake is 137 nm. The thicknesses of all the FGT flakes are extracted using the same procedure.

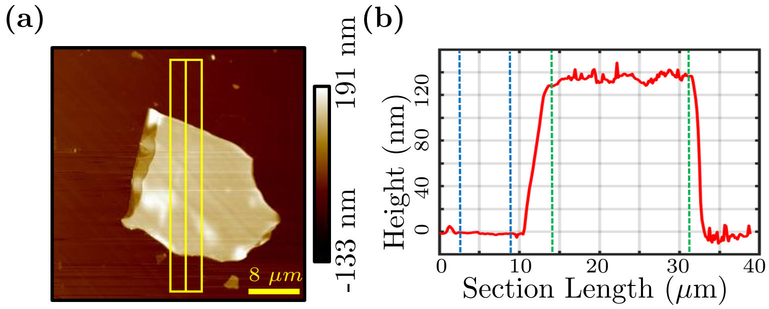

Figure S2: (a) Surface topography image of an FGT flake produced by AFM in the tapping mode. (b) The average step-height profile for the selected region shown (yellow) in (a). Here vertical blue and green dashed lines are used to select the length elements (corresponding to an area enclosed by the yellow box in (a)) to estimate the flake height.

S3. Magnetization Extraction

Steps followed to extract magnetic field and magnetization images from the pixel-wise ODMR data:

1. Pixel-wise, the experimental ODMR data were fitted, and a raw 2D magnetic field image was extracted.

2. The average external bias magnetic field (B_z^{ext}) was extracted from the raw image by removing the flake region.

3. B_z^{ext} was subtracted from the raw magnetic image to obtain the magnetic field (B_z) image of the flake.

4. Further, to compute magnetization, the background noises in the magnetic image of the flake were also removed.

5. Through reverse propagation the OOP magnetization (M_z) of the flake was reconstructed from B_z.[1,2]

6. The average magnetization and standard deviation were obtained from the reconstructed M_z.

S4. Temperature Control

A temperature controller (Lightwave LDT-5910B) was used to control the temperature during temperature-dependent measurements. A 14.184 kΩ thermistor, as the temperature probe, was placed on the diamond surface in the vicinity of flakes. A Peltier-based thermoelectric cooler (Marlow Industries Inc., Model: RC3-6(58954)) was placed on top of the diamond surface for cooling/heating along with an aluminum heat sink. A thermal paste (Wakefield Solutions, Type: 120 Silicon) was applied between the diamond surface, thermistor, and cooler for efficient thermal conductivity. During the measurements, the temperature fluctuations were $\sim\pm 0.1$ K.

S5. Thin Flake Chemical Degradation

In general, iron tends to oxidize when exposed to oxygen. The FGT flakes being composed of iron tend to oxidize when exposed to the air environment. The oxidized FGT layer is no

longer ferromagnet and remains on top of the non-oxidized layer, acting as a protective shield for the remaining non-oxidized flake. For thicker layers around 50 nm, the non-oxidized layer retains reasonable thickness and can be measured easily for 3-4 days or longer depending upon the thickness. However, thinner flakes can oxidize completely, making measurement challenging. We also observed the same. A thin flake observed and located in the optical microscope is measured directly after the exfoliation and transferred onto the diamond Fig.S5(a). The same flake was measured after around 12 hours, and we observed that it had decayed, Fig.S5(b). To confirm the flake measured is thin AFM measurement was performed, Fig.S5(c). The AFM analysis reveals its average thickness of around 26 nm, Fig.S5(d). This

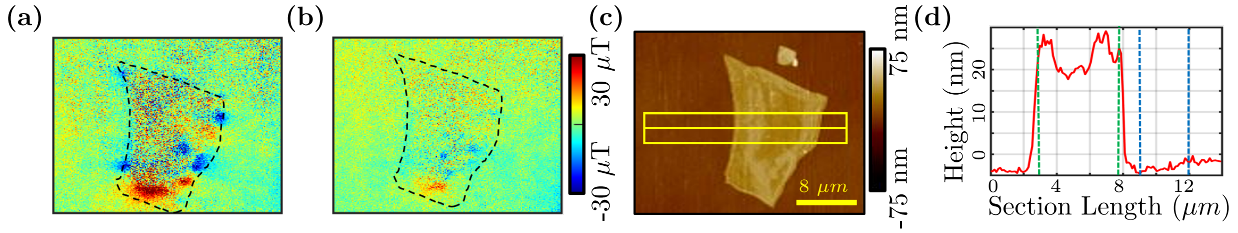

Figure S5: Magnetic images of a 26 nm thin FGT flake measured (a) immediately after the transfer onto the diamond surface. (b) 12 hours after the transfer onto the diamond surface. (c) AFM topography image of the FGT flake (d). The average step-height profile was obtained from the yellow rectangle shown in (c).

oxidation of FGT flakes in an oxygen environment makes it hard to measure thin flakes ($<$20 nm). To prevent oxidation, one can either encapsulate the FGT flake with hexagonal boron nitride flake or evaporate a few nanometers of gold or platinum. Here for measuring thin flakes, the flakes are transferred on the diamond surface in the glove box (MBraun glove box) connected to an evaporator (VST glove box evaporator) and sent directly for evaporating gold $\sim$5 nm to prevent it from oxidation.

S6. Temperature Dependent Magnetic Imaging

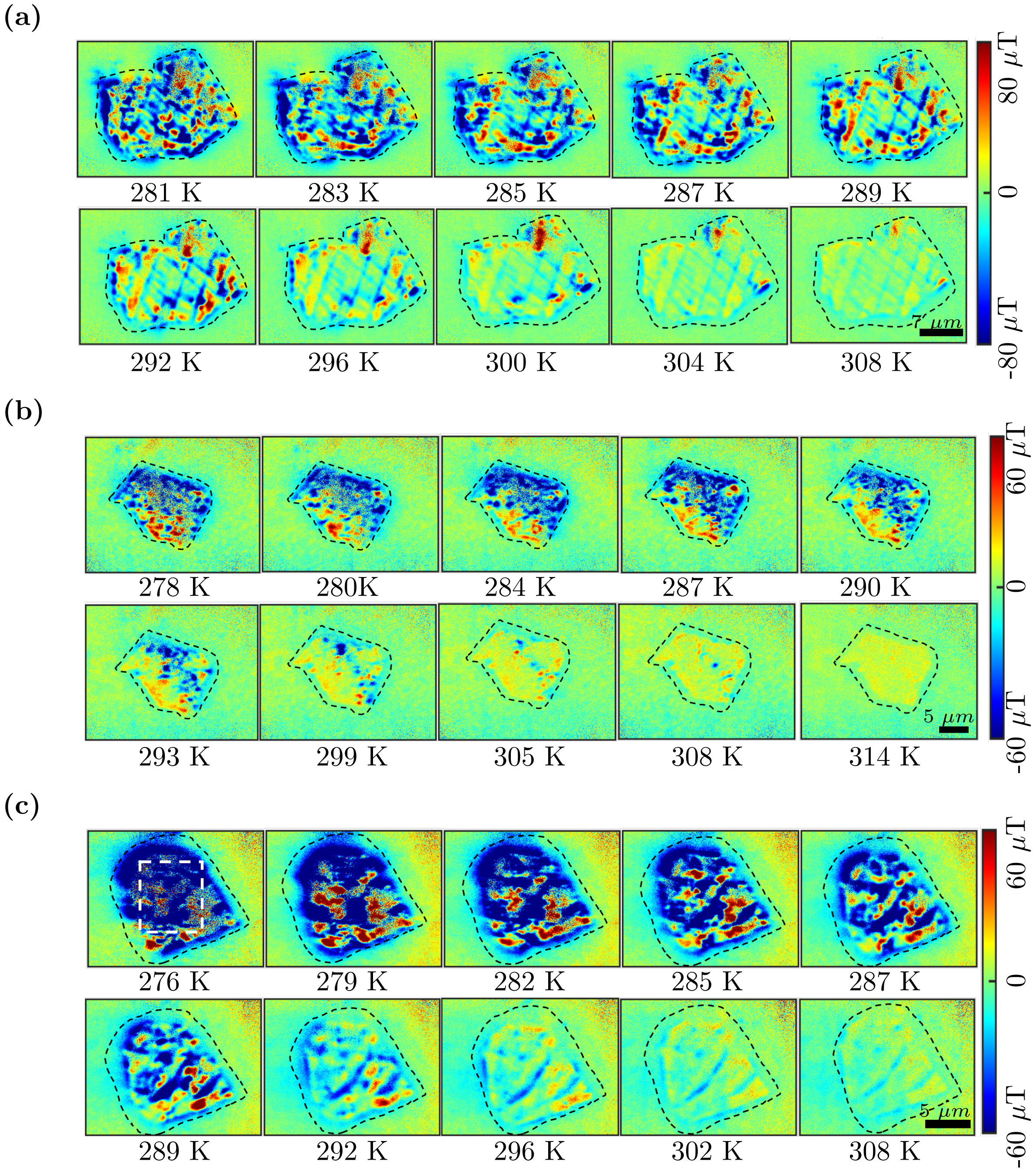

Figure S6: (a)-(c) Temperature dependent magnetic images of several FGT flakes. (c) The white dashed rectangle represents the region considered for the autocorrelation map depicted in Fig. 6(b) of the main article.

S7. Wide-field versus Scanning NV Spectrometer Magnetic Imaging

In order to understand the quantitative difference observed in the computed stray magnetic fields, in Fig.4(a),(b) and Fig.7(a) of the main article, magnetic imaging experiments were repeated on wide-field and scanning NV magnetometers.

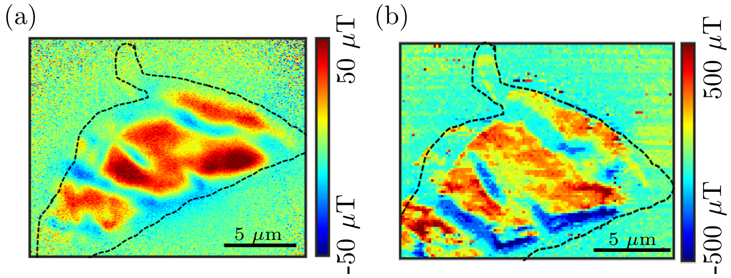

Figure S7: Magnetic image obtained from (a) Wide-field NV spectrometer (b) Scanning NV spectrometer.

Results are shown in the figure above, S7. These measurements are qualitatively similar, though quantitative differences can be observed in the stray fields. One possible reason could be the difference in the standoff distance of the NV sensors from the FGT flake.

S8. Temperature Dependent Magnetization

Below are the extracted magnetization plots based on the procedure explained in Sec.-S3. The stray fields shown in Fig. 4(a) and (b) of the main article were utilized to obtain these magnetization plots.

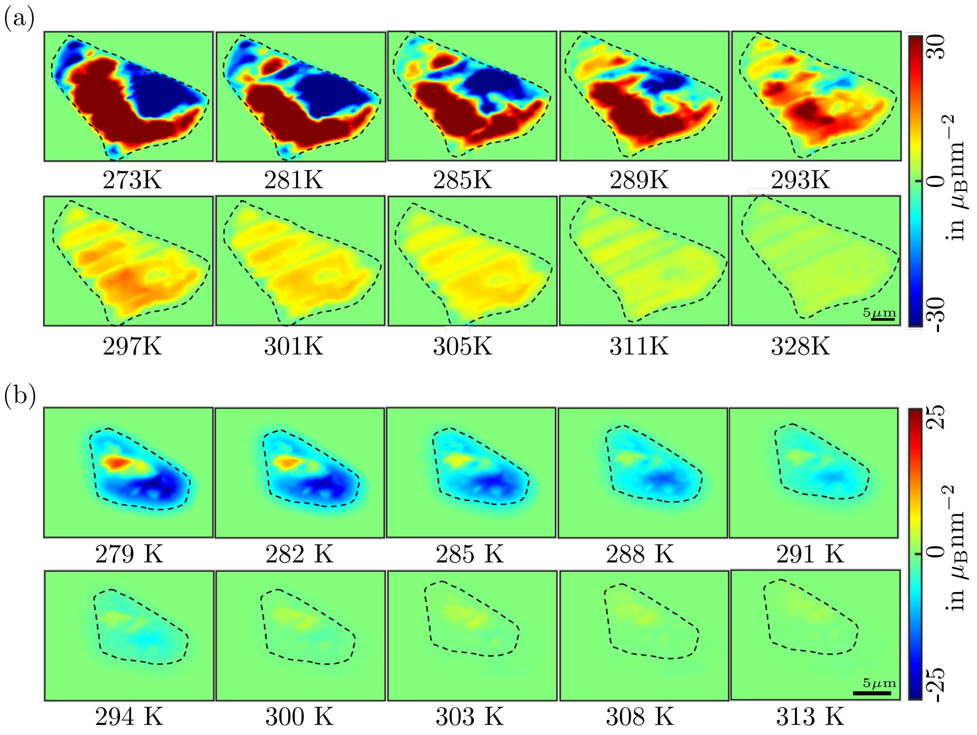

Figure S8: Temperature dependent magnetization plots.

S9. The EDXS Analysis of FGT Flakes

The top panel of Fig. S9 shows SEM images of several FGT flakes. EDXS analysis was performed on these flakes to examine compositional modulations similar to those presented in Fig. 8(a)–(c) of the main article. The green ladder visible in the SEM images marks the observation points where EDXS measurements were carried out, comprising a total of fifty data points. It is noteworthy that the stripe-like features in the SEM images are only faintly visible under the present imaging conditions; these features are typically more discernible at an electron beam energy of 10 keV. The lower panel displays the corresponding elemental modulation, primarily in iron (Fe), oxygen (O), and germanium (Ge). Interestingly, the regions where the stripes are more clearly visible in the SEM images coincide with stronger modulations observed in the EDXS profiles. Repeated measurements across multiple flakes confirmed that these stripe-like features are reproducible and consistently observed in samples from different batches.

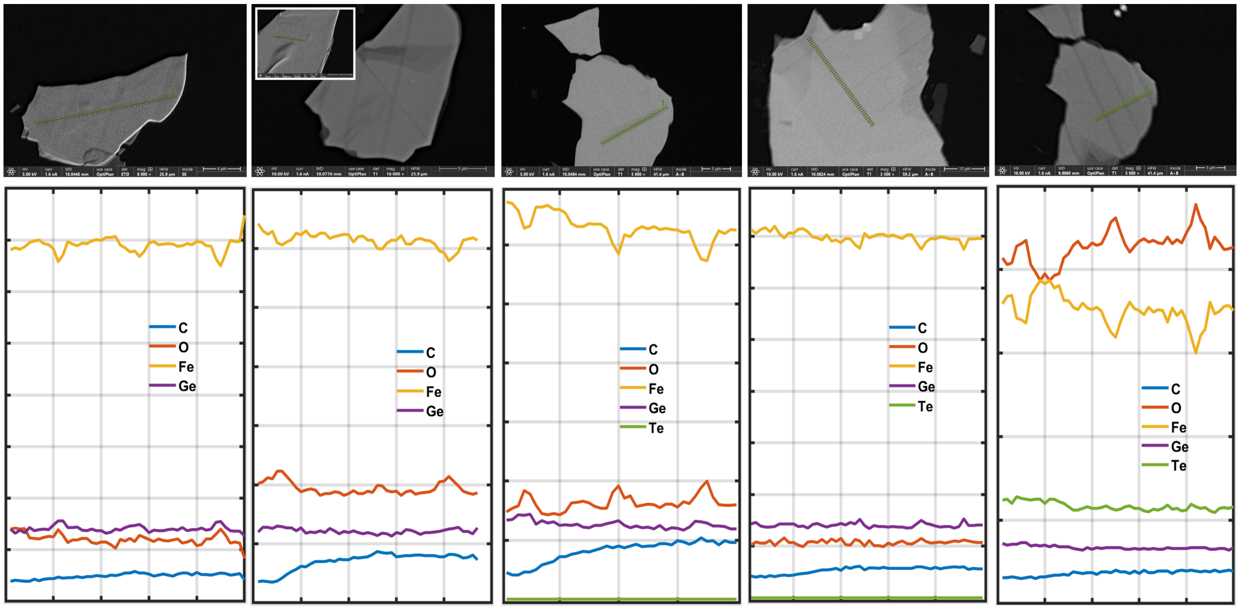

Figure S9: Top panel are the SEM images of several FGT flakes. Respective EDXS results are presented the panel below.

References

(1) Tan, S.; Ma, Y. P.; Thomas, I.; Wikswo, J. Reconstruction of two-dimensional magnetization and susceptibility distributions from the magnetic field of soft magnetic materials. *IEEE Trans. Magn.* **1996**, *32*, 230–234.

(2) Thiel, L.; Wang, Z.; Tschudin, M. A.; Rohner, D.; Gutiérrez-Lezama, I.; Ubrig, N.; Gibertini, M.; Giannini, E.; Morpurgo, A. F.; Maletinsky, P. Probing magnetism in 2D materials at the nanoscale with single-spin microscopy. *Science* **2019**, *364*, 973–976.
